# Supplementary material for: Regulation of the COPII secretory machinery via focal adhesions and extracellular matrix signaling
Source: J Cell Biol. 2022 Jul 13;221(8):e202110081. doi: 10.1083/jcb.202110081 (PMC9284426; doi:10.1083/jcb.202110081)
Supplement: SourceData F3 — contains original blots for Fig. 3. [file JCB_202110081_SourceDataF3.pdf]

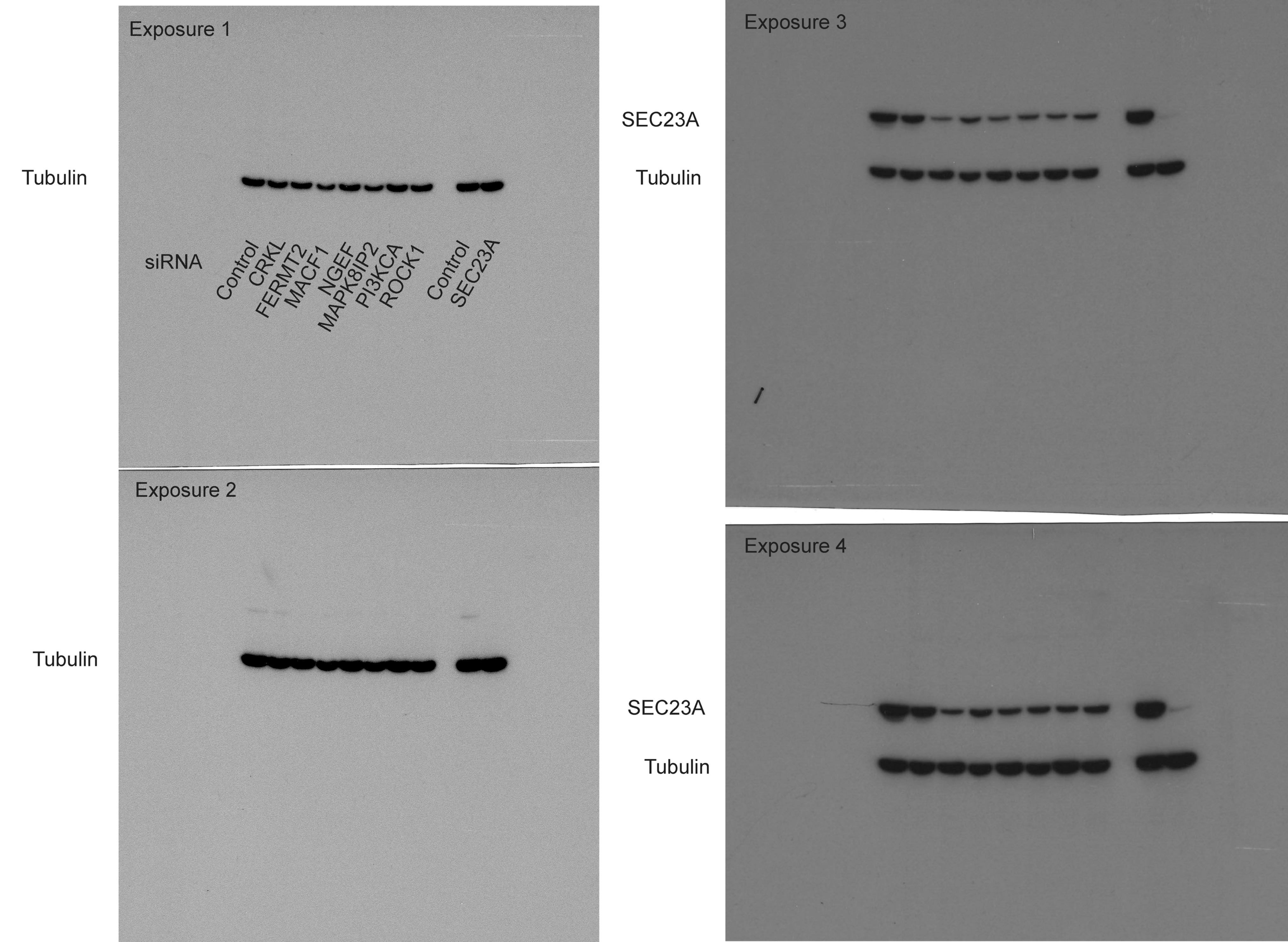
Exposure 1
Tubulin
siRNA
Control
CRKL
FERMT2
MACF1
NGEF
MAPK8IP2
PI3KCA
ROCK1
Control
SEC23A
Exposure 2
Tubulin
Exposure 3
SEC23A
Tubulin
Exposure 4
SEC23A
Tubulin
