# Supplementary material for: Regulation of the COPII secretory machinery via focal adhesions and extracellular matrix signaling
Source: J Cell Biol. 2022 Jul 13;221(8):e202110081. doi: 10.1083/jcb.202110081 (PMC9284426; doi:10.1083/jcb.202110081)
Supplement: SourceData F5 — contains original blots for Fig. 5. [file JCB_202110081_SourceDataF5.pdf]

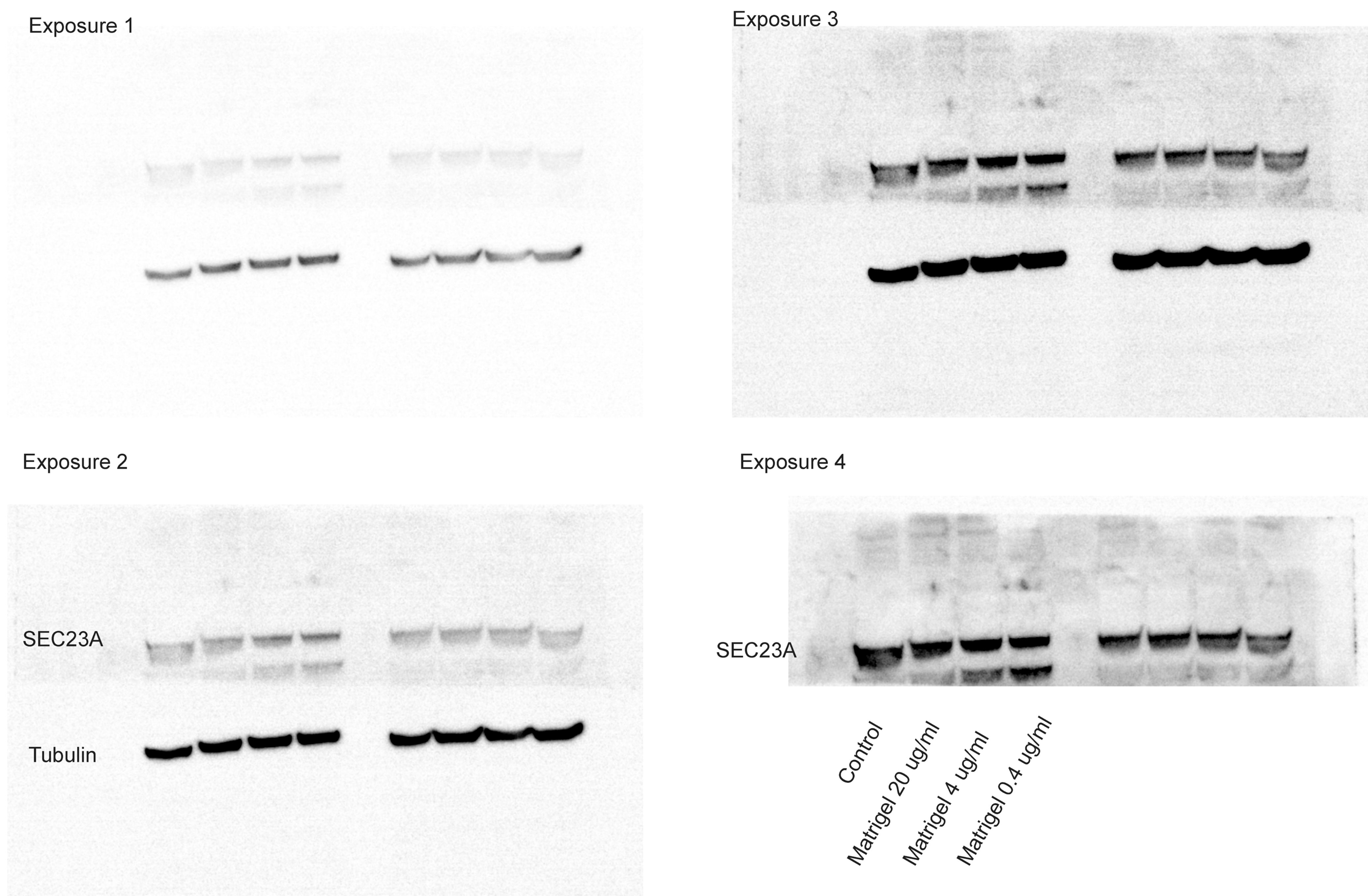
Exposure 1
Exposure 3
Exposure 2
SEC23A
Tubulin
Exposure 4
SEC23A
Control
Matrigel 20 ug/ml
Matrigel 4 ug/ml
Matrigel 0.4 ug/ml
