# Supplementary figures and images for: Regulation of the COPII secretory machinery via focal adhesions and extracellular matrix signaling
Source: J Cell Biol. 2022 Jul 13;221(8):e202110081. doi: 10.1083/jcb.202110081 (PMC9284426; doi:10.1083/jcb.202110081)

Exposure 2

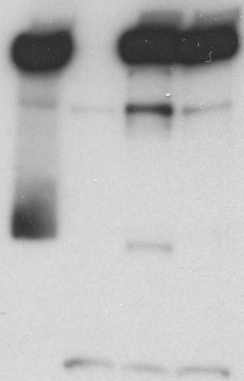

Exposure 4

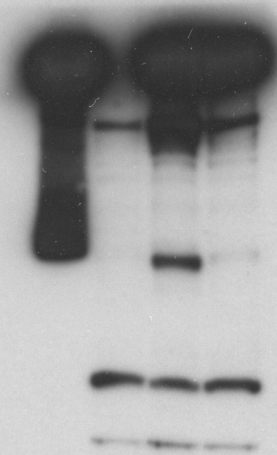

MACF1

SEC23B

Exposure 1

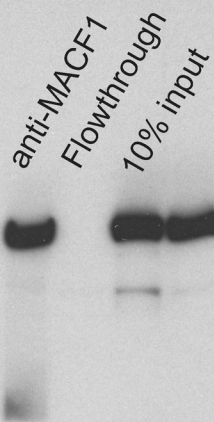

MACF1

Exposure 3

105 -  
120 -  
85 -  
65 R -

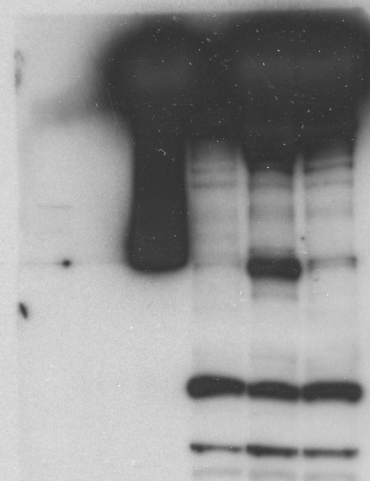

MACF1

SEC23B

Supplement: SourceData FS1 — contains original blots for Fig. S1. [file JCB_202110081_SourceDataFS1.pdf]
